# ﻿Resurrection and redescription of *Clepsinepallida* Verrill, 1872 (Hirudinida, Glossiphoniidae) with a phylogeny of the genus *Alboglossiphonia*

**DOI:** 10.3897/zookeys.1127.86004

**Published:** 2022-11-02

**Authors:** William E. Moser, Dennis J. Richardson, Charlotte I. Hammond, Lourdes Rojas, Eric Lazo-Wasem, Anna J. Phillips

**Affiliations:** 1 Smithsonian Institution, National Museum of Natural History, Department of Invertebrate Zoology, Museum Support Center MRC 534, 4210 Silver Hill Road, Suitland, MD 20746, USA National Museum of Natural History Suitland United States of America; 2 School of Biological Sciences, Quinnipiac University, 275 Mt. Carmel Avenue, Hamden, CT 06518, USA Quinnipiac University Hamden United States of America; 3 Division of Invertebrate Zoology, Peabody Museum of Natural History, Yale University, P.O. Box 208118, New Haven, CT 06520, USA Yale University New Haven United States of America; 4 Smithsonian Institution, National Museum of Natural History, Department of Invertebrate Zoology, 10th St and Constitution Ave, NW, Washington, DC 20560-0163, USA National Museum of Natural History Washington United States of America

**Keywords:** *Alboglossiphoniaheteroclita*, Clitellata, *Glossiphonia*, *Glossiphoniaswampina*, Glossiphoniiformes, leech, Rhynchobdellida

## Abstract

*Alboglossiphoniapallida* (Verrill, 1872) **comb. nov.** is resurrected and redescribed based on morphological and molecular data from specimens of the type locality (New Haven County, Connecticut, USA) that demonstrate it is distinct from North American *Alboglossiphoniaheteroclita*, European *Alboglossiphoniaheteroclita*, and *Alboglossiphoniapapillosa*. *Alboglossiphoniapallida* is characterized by having dark chromatophores on the dorsal surface arranged lateral to patrilaterally and medially as a thin line or interrupted thin line along with three pairs of eye spots (with the first pair closest together), six pairs of crop ceca, and a united gonopore. Additional sampling of specimens of the genus *Alboglossiphonia* is needed to understand its phylogeny especially as many species have not been collected since their description.

## ﻿Introduction

The species concept of *Alboglossiphoniaheteroclita* (Linnaeus, 1761) has become very heterogenous over time through a combination of formal synonymy and informal accumulation of diagnostic morphological characters. Other species of the genus *Alboglossiphonia* exhibit similar taxonomic confusion, including *Alboglossiphoniahyalina* (O.F. Müller, 1774), *Alboglossiphoniainflexa* (Goddard, 1908), *Alboglossiphonianovaecaledoniae* (Johansson, 1918), *Alboglossiphoniapapillosa* (Braun, 1805), and *Alboglossiphoniastriata* (Apáthy, 1888) (Lukin 1976; Nesemann and Neubert 1999; Govedich 2001; Kaygorodova et al. 2014; Klass et al. 2018; Bolotov et al. 2019).

*Hirudoheteroclita* was originally described from Europe by Linnaeus (1761) and is characterized by the possession of six eye spots and a translucent body with black spots. Moquin-Tandon (1846) transferred *H.heteroclita* to the genus *Glossiphonia* Johnson, 1816. Carena (1820) and Blanchard (1894) stated that this species was very rare. Based upon pigmentation and the comparative distance between the first pair versus the second and third pair of eye spots, Lukin (1976) erected the subgenus Alboglossiphonia containing Glossiphonia (Alboglossiphonia) heteroclita. Klemm (1982) raised *Alboglossiphonia* to the genus rank, creating the combination *Alboglossiphoniaheteroclita* as the type species.

**Figure 1.**
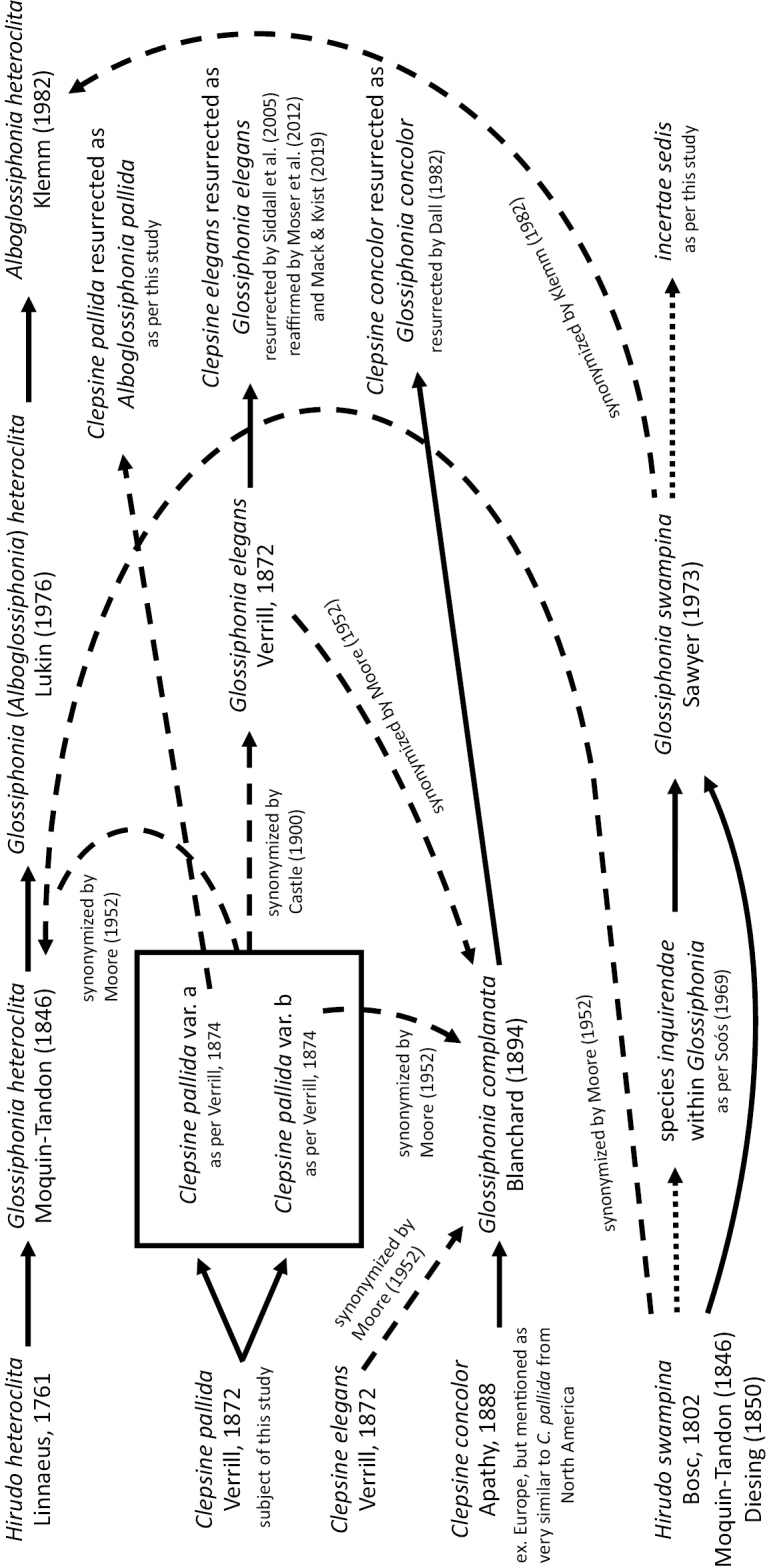
Schematic representation of the taxonomic history of *Alboglossiphoniapallida* (Verrill, 1872). Solid line (taxonomic act), dashed line (synonymization), and dotted line (*incertum*).

In North America, Verrill (1872) described *Clepsinepallida* based on individuals from the West River of New Haven, Connecticut (Fig. 1). *Clepsinepallida* is characterized by the possession of six eyes and a pale body with scattered black specks and a median light line interrupted by a row of small black spots (Verrill 1872). Verrill (1874) updated the species concept by describing *C.pallida* Verrill, 1872 as *Clepsinepallida* var. a, and described *Clepsinepallida* var. b from New Haven, Connecticut and Colorado, respectively. As described by Verrill (1874), *Clepsinepallida* var. b is very similar to the North American *Glossiphoniaelegans* (Verrill, 1872), a leech species resurrected by Siddall et al. (2005) and subsequently reaffirmed by Moser et al. (2012) and Mack and Kvist (2019).

In describing *Glossiphoniaconcolor* from Europe, Apáthy (1888) mentioned that the species was very similar to *C.pallida* in North America, but he did not indicate which of Verrill’s varieties (var. a or var. b) was the most similar. In European studies, Blanchard (1894) considered *G.concolor* to be a simple variety of *Glossiphoniacomplanata* (Linnaeus, 1758), thus inferring similarity of *C.pallida* to *G.complanata* (including *Glossiphoniaelegans* (Verrill, 1872) that was considered a synonym to *G.complanata* at that time). Castle (1900) synonymized *C.pallida* with *Glossiphoniaelegans* (Verrill, 1872) while simultaneously recognizing *G.heteroclita* from the vicinity of Cambridge, Massachusetts. *Clepsinepallida* was subsequently ignored until Moore (1952) severed the association of *C.pallida* with *G.complanata* and determined *C.pallida* as a junior synonym of *Glossiphoniaheteroclita* (Linnaeus, 1761) (Fig. 1). However, Soós (1969) caused further confusion by listing *Clepsinepallida* as a synonym of *Glossiphoniacomplanata* in his key and comprehensive list of all the species of the family Glossiphoniidae.

*Hirudoswampina* was described by Bosc (1802) as abundant in the swamps of “Carolina” and attached to turtles or frogs. As described, *H.swampina* has five eye spots, a rough dorsum with green varied with brown, and the head, margins, and the posterior are spotted with white. The species description was updated by Moquin-Tandon (1846) and Diesing (1850). Although the species description indicated *H.swampina* possessed five eye spots, the redescriptions indicated that “five?” (Moquin-Tandon 1846) or six (Diesing 1850) eye spots were found, a character also present in several species of *Alboglossiphonia* and *Glossiphonia*. However, the rough dorsum, green/brown coloration, and attachment to turtles and frogs indicate that it is more similar to a species in the genus *Placobdella*, as species of *Alboglossiphonia* and *Glossiphonia* feed on invertebrates. *Placobdellahollensis* (Whitman, 1892) has up to five pairs of accessory “eyes” and has been found in North Carolina (Moser et al. 2017), and Sawyer (2021) stated that some adult individuals of *Placobdellamultilineata* from North Carolina have pigment patterns that resemble multiple eye spots (accessory eyes). In comparison with the drawing and description of Bosc (1802), *H.swampina* could just as likely have represented an undesignated species of *Placobdella* as it could have represented *Glossiphonia* or *Alboglossiphonia*. It is likely that Bosc (1802) used the name *H.swampina* to describe a suite of species presently recognized as belonging to *Placobdella*. It is clear from Bosc (1802) that *H.swampina* referred to a leech parasitic on turtles and frogs. Additionally, it is assumed that the description of Bosc (1802) was based on specimens from the “Carolinas” of the United States. However, Moquin-Tandon (1846) indicated that *H.swampina* parasitized turtles and frogs in the marshes of South America. Adding credence to this understanding, there are references to “Carolina” in Argentina, Brazil, and Surinam. Soós (1969) listed *H.swampina* as a *species inquirenda* in the genus *Glossiphonia*.

**Figure 2.**
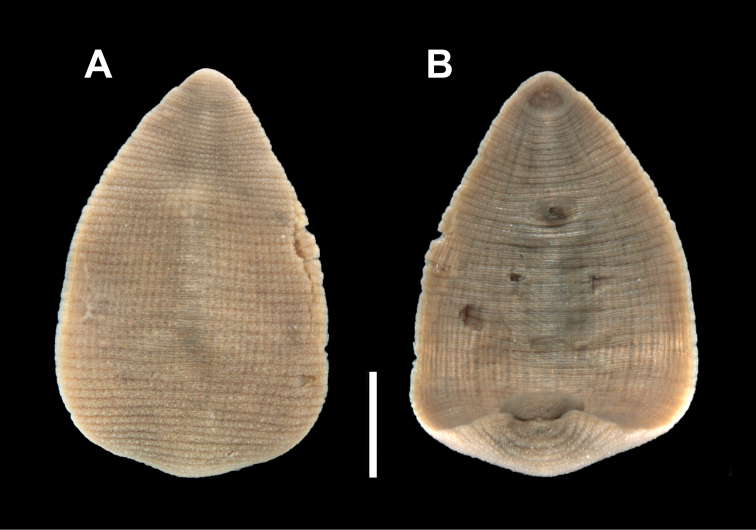
Holotype specimen of *Clepsinepallida* Verrill, 1872 (YPM IZ 000253) **A** dorsal surface **B** ventral surface. Scale bar: 1 mm.

Ignoring the similarities of *H.swampina* to the genus *Placobdella*, Moore (1952) declared *Clepsineswampina* as a junior synonym of *G.heteroclita* (= *A.heteroclita*). Later, Sawyer (1973) published a rediscovery of *Glossiphoniaswampina* (Bosc, 1802) from two localities in the coastal plain of South Carolina and deposited a neotype in the National Museum of Natural History (USNM 47122), Smithsonian Institution. Sawyer (1973) stated that *G.swampina* is distinct from the unpigmented, translucent *G.heteroclita*, because *G.swampina* has four to seven mid-dorsal pigment bars. However, such a pigmentation pattern also occurs in *C.pallida* (Fig. 2). Additional specimens of *G.swampina* were found in the coastal plain of North Carolina by Sawyer and Shelley (1976). Klemm (1976) suggested that *G.swampina* is a color variant of *G.heteroclita*, and after examining specimens from Quebec and Maryland, Klemm (1982) declared *G.swampina* a junior synonym of *A.heteroclita*.

In recent phylogenetic studies, Trontelj et al. (1999) used *A.heteroclita* from Italy and Apakupakul et al. (1999), Light and Siddall (1999), and Siddall et al. (2005) used *A.heteroclita* from Michigan, Jueg (2008) used *A.heteroclita* from Germany and Michigan, and Bolotov et al. (2019) used *A.heteroclita* from Michigan and *A.papillosa* from Russia as a basis for molecular studies. However, *A.heteroclita* from Europe and *A.heteroclita* from North America have not been compared to *A.papillosa* nor to specimens of *C.pallida* in a molecular analysis.

The convoluted history of this assemblage is given in Fig. 1. In this study, we provide a molecular comparison of contemporary specimens that are morphologically consistent with the *C.pallida* of Verrill (1872) and *C.pallida* var. a of Verrill (1874) collected from the type locality of West River, Connecticut to specimens identified as *A.heteroclita* from Michigan, USA and from Germany, providing the basis for a redescription, resurrection, and molecular characterization of *C.pallida*.

## ﻿Materials and methods

### ﻿Collection of leeches and morphological analysis

During the course of a survey of the leech fauna of south-central Connecticut, individuals matching the description of *Clepsinepallida* Verrill, 1872 were collected by hand from submerged substrate in the West River, New Haven, New Haven County, the type locality of *C.pallida*. Specifically, collections were made from the West River at Konolds Pond (41°20'52.1"N, 72°58'41.6"W) and Whalley Avenue Bridge (41°19'30.13"N, 72°57'26.76"W) south to the “Duck Pond” (41°18'51.30"N, 72°57'21.75"W) as illustrated on page 12 of Shumway and Hegel (1990) and Clark’s Pond (41°24'47.9"N, 72°53'46.8"W) between May 2008 and September 2009, and later in September 2020 and July 2021. A collection was also made from Sturges Pond (41°11'50"N, 73°18'2"W), Larsen Sanctuary, Fairfield County Connecticut on 27 July 2021. Specimens were relaxed, examined, and fixed as described by Moser et al. (2006). Several specimens were pressed, stained with Semichon’s acetocarmine and mounted in Canada Balsam for examination by light microscopy according to techniques outlined by Richardson (2006), as modified by Richardson and Barger (2006). Specimens were examined using an Olympus SZX16 dissecting microscope and were photographed with a Zeiss Stemi 2000-CS macroscope fitted with a Q-Capture 5.0 RTV Micropublisher camera. Images were acquired at different focal levels and the resulting stacks rendered with Helicon Focus 7 Pro to make an extended focus image. Post-processing was done using Adobe Photoshop CC 2015. Terminology for plane shapes follows Clopton (2004). Specimens were deposited in the Peabody Museum of Natural History (**YPM**), Yale University, New Haven, Connecticut, USA and the National Museum of Natural History (**USNM**), Smithsonian Institution, Washington, District of Columbia, USA.

### ﻿DNA and phylogenetic analysis

Molecular analyses were conducted on newly collected material according to Richardson et al. (2010) as follows: DNA was isolated from the caudal suckers of five individual leeches (YPM IZ 058354, YPM IZ 062698, YPM IZ 109351–109353) with the DNeasy Blood & Tissue Kit from Qiagen (cat. no. 69504), following the protocol given for the purification of total DNA from animal tissues (spin-column). For the proteinase K treatment step, tissue samples were lysed overnight at 56 °C. DNA was eluted from the spin columns with 150 µl of buffer.

Polymerase chain reactions (PCR) were prepared using the Illustra PuRe Taq Ready-To-Go PCR beads from GE Health Care (cat. no. 27-9559-01). Primers were purchased from Invitrogen and were comprised of two primers each for cytochrome c oxidase subunit I (COI) as specified by Folmer et al. (1994) and Light and Siddall (1999). Specifically, the COI primers were LCO1490 (5'-GGTCAACAAATCATAAAGATATTGG-3') and HCO2198 (5'-TAAACTTCAGGGTGACCAAAAAATCA-3'). Final volume of PCR reactions was 25 µl with 2 µl of leech genomic DNA added per reaction. DNA was amplified under the following PCR conditions: 94 °C for 5 min; 35 cycles of (94 °C for 30 s, 50 °C for 30 s, 72 °C for 45 s); 72 °C for 7 min. Following PCR, samples were cleaned up using a QIAquick PCR purification kit from Qiagen (cat. no. 28104).

Purified PCR products were sequenced using the HCO2198 and LCO1490 primers for the COI products by the W.M. Keck Foundation Biotechnology Resource Laboratory at Yale University. DNA sequences were edited and assembled using Geneious Prime (v. 2020.1.2, Biomatters Ltd.). Novel sequences were deposited in GenBank (Benson et al. 2018; Table 1). Comparable sequence data for seven recognized *Alboglossiphonia* species (24 sequences), sequences identified as *Alboglossiphonia* sp. (three individuals), *Glossiphoniacomplanata* (two individuals), and *Glossiphoniaelegans* (two individuals) were downloaded from GenBank (Table 1). Additionally, five sequences identified as *A.heteroclita* were downloaded from BOLD (Ratnasingham and Hebert 2013; Table 1). The COI sequences were aligned using the MAFFT multiple sequence alignment plug-in for Geneious Prime (Katoh and Standley 2013) with default settings, checked by eye for gaps, and the sequences were translated to amino acids to assess sequence quality. Uncorrected pairwise sequence distances were calculated using Geneious Prime.

**Table 1.** Species, collection locality, museum catalog number, and Genbank accession information or BOLD for sequences included in this study.

Species	State/province	Country	Location	Catalog number /citation	GenBank or BOLD#
*Alboglossiphoniaiberica*	Huelva	Spain		8789, Jueg 2008	N/A
*Alboglossiphoniaquadrata*		Namibia		Siddall et al. 2005	AY962455
*Alboglossiphoniaheteroclita*		Germany		9195, Jueg 2008	N/A
*Alboglossiphoniaheteroclita*	Michigan	USA		Apakupakul et al. 1999	AF116016
*Alboglossiphoniaheteroclita*	Michigan	USA		BSC-160.1, SUNY Buffalo State	ANNMO802-20
*Alboglossiphoniaheteroclita*	Michigan	USA		BSC-160.2, SUNY Buffalo State	ANNMO803-20
*Alboglossiphoniaheteroclita*	Michigan	USA		BSC-160.6, SUNY Buffalo State	ANNMO807-20
*Alboglossiphoniaheteroclita*	Wisconsin	USA		BSC-160.3, SUNY Buffalo State	ANNMO804-20
*Alboglossiphoniaheteroclita*	Wisconsin	USA		BSC-160.4, SUNY Buffalo State	ANNMO805-20
*Alboglossiphoniapallida*	Connecticut	USA	Konolds Pond, West River	YPM IZ 058354, this study	ON738431
*Alboglossiphoniapallida*	Connecticut	USA	Konolds Pond, West River	YPM IZ 109351, this study	ON738432
*Alboglossiphoniapallida*	Connecticut	USA	Konolds Pond, West River	YPM IZ 109352, this study	ON738433
*Alboglossiphoniapallida*	Connecticut	USA	Konolds Pond, West River	YPM IZ 109353, this study	ON738434
*Alboglossiphoniapallida*	Connecticut	USA	Clarks Pond	YPM 062698, this study	ON738435
*Alboglossiphoniapapillosa*	Siberia	Russia	Lake Gusinoe	Kaygorodova et al. 2014	KM095100
*Alboglossiphoniapapillosa*	Siberia	Russia	Lake Gusinoe	Kaygorodova et al. 2014	KM095101
*Alboglossiphoniapapillosa*	Siberia	Russia	Lena River basin	RMBH Hir13/3, Klass et al. 2018	MH286269
*Alboglossiphoniapapillosa*	Siberia	Russia	Lena River basin	RMBH Hir13/4, Klass et al. 2018	MH286270
*Alboglossiphoniapapillosa*	Siberia	Russia	Lena River basin	RMBH Hir13/5, Klass et al. 2018	MH286271
*Alboglossiphoniapapillosa*	Siberia	Russia	Lena River basin	RMBH Hir13/2, Klass et al. 2018	MH286268
*Alboglossiphoniapapillosa*	Siberia	Russia	Lena River basin	RMBH Hir13/1, Klass et al. 2018	MH286267
*Alboglossiphonia* sp. 2		Myanmar		RMBH HIR58/2 Bolotov et al. 2019	MN295404
*Alboglossiphonia* sp.	Victoria	Australia	Melbourne	MRD16Gloss2, Carew et al. 2018	MG976199
*Alboglossiphonialata*	Primorsky Krai	Russia		RMBH HIR58/1, Bolotov et al. 2019	MN295414
*Alboglossiphonialata*		South Korea		RMBH HIR113/4, Bolotov et al. 2019	MN393286
*Alboglossiphonialata*		South Korea		RMBH HIR103/5, Bolotov et al. 2019	MN393275
*Alboglossiphonialata*		South Korea		RMBH HIR110/5, Bolotov et al. 2019	MN393279
*Alboglossiphonialata*		South Korea		RMBH HIR113/3, Bolotov et al. 2019	MN393284
*Alboglossiphonialata*		South Korea		RMBH HIR112/1, Bolotov et al. 2019	MN393281
*Alboglossiphonialata*		South Korea		RMBH HIR114/12, Bolotov et al. 2019	MN393288
*Alboglossiphonialata*		South Korea		RMBH HIR111/22, Bolotov et al. 2019	MN393280
*Alboglossiphonialata*		South Korea		RMBH HIR114/1, Bolotov et al. 2019	MN393287
*Alboglossiphonialata*		South Korea		RMBH HIR109/1, Bolotov et al. 2019	MN393276
*Alboglossiphonialata*		South Korea		RMBH HIR110/32, Bolotov et al. 2019	MN393277
*Alboglossiphonialata*		South Korea		RMBH HIR113/32, Bolotov et al. 2019	MN393285
*Alboglossiphoniaweberi*	Hawaii	USA		Siddall et al. 2005	AY962453
*Alboglossiphonia* sp.		South Korea		HJK-2020, Kwak et al. 2021	MN503262
*Glossiphoniacomplanata*		United Kingdom		Light and Siddall 1999	AY047321
*Glossiphoniacomplanata*	Mecklenburg-Vorpommern	Germany		Trajanovski et al. 2010	HM246608
*Glossiphoniaelegans*	Connecticut	USA	West River	Moser et al. 2012	JQ073858
*Glossiphoniaelegans*	Connecticut	USA	West River	Moser et al. 2012	JQ073860

The best partitioning scheme was tested using ModelFinder within IQ-TREE (Kalyaanamoorthy et al. 2017) using the -*m MF+MERGE* command, as well as estimation of substitution models by codon position, resulting in the following models as best fit by partition by the Bayesian information criterion: first codon position = F81+F, second codon position = TN+F+I, and third codon position = K3Pu+F+G4. A maximum likelihood (ML) analysis was performed with IQ-TREE v. 1.6.12 (Nguyen et al. 2015), using the models suggested for each unlinked partition, the -*spp* command to allow each partition to have its own evolutionary rate, and 1,000 ultrafast bootstrap (UFBOOT2) approximations (Hoang et al. 2018). *Glossiphoniacomplanata* (AY047321 and HM246608) and *Glossiphoniaelegans* (JQ073858 and JQ73860) served as outgroups. FigTree v. 1.4.4 (Rambaut 2018) was used to visualize trees that were then edited with Adobe Illustrator Creative Cloud (https://www.adobe.com).

## ﻿Results and discussion

### ﻿Morphological analysis

Examination of the type series of *Clepsinepallida* (YPM IZ 00253) revealed a single specimen (Fig. 2). The more than 150-year-old holotype specimen is remarkably well preserved, but the pigmentation has faded and the eye spots are no longer discernible. The dorsal surface is smooth and there is a united gonopore. The original YPM Invertebrate Zoology Annelida ledger entry for YPM IZ 00253 indicates a single specimen of *Clepsinepallida* V. collected from West River, New Haven, Connecticut on 6 June 1871 and is labeled as type.

In further examination of the YPMAnnelida Ledger, *Clepsinepallida* var. a and var. b of Verrill (1874) had not been assigned a catalog number. No specimens of *Clepsinepallida* var. a were found, and Verrill’s (1874) account likely refers only to the holotype specimen, YPM IZ 00253. In Verrill (1874), *Clepsinepallida* var. b came from Colorado (US Geological and Geographic Survey of the Territories, i.e. Hayden’s expedition) and again, Colorado (lake near Long’s Peak, 9,000 feet elevation, Hayden’s expedition, 1873). In the YPM uncataloged leech holdings, two lots were recently discovered that are likely the syntypes of *Clepsinepallida* var. b. One lot (now YPM IZ 106808) had an original label in Sidney Smith’s handwriting that reads “Colorado Mts. & Plains, 1873” and another label in A.E. Verrill’s handwriting as “*Clepsinepallida* var. b Colorado Mts. & Plains Haydens Exp. 1873.” On the second label, *pallida* has been crossed out and “*elegans*” has been written in Verrill’s handwriting, indicating an updated identification as *Clepsineelegans*. The single specimen (YPM IZ 106808) is in very good condition and morphologically consistent with *Glossiphoniaelegans* (six eye spots; pair of dark paramedial lines; two pair of metameric white dots).

The second lot (now YPM IZ 106809) had a label in J. Percy Moore’s handwriting as “*Clepsinepallida* Verrill, near Longs Peak, 9000 ft, Haydens Exp” with a reidentification as *Glossiphoniacomplanata* (Linnaeus) and a label written by former Yale Curator of Invertebrate Zoology Willard Hartman as “*Glossiphoniacomplanata* (Linn) Lake near Long’s Peak, 9000 ft., Hayden’s Expedition, 1873; Verrill’s Ident: *Clepsinepallida*” – no Verrill-era label was found. This information matches Verrill (1874) of *Clepsineelegans* var. b. YPM IZ 106809 containing three specimens of average condition which have likely dried out and subsequently been rehydrated without benefit of a restorative surfactant. Two of the specimens are morphologically consistent with *Glossiphoniaelegans* (six eye spots and pair of paramedial dark lines). The third specimen is smaller and difficult to discern.

Sawyer (1973) designated a neotype (USNM 47122) and an additional specimen (USNM 51436) of *Glossiphoniaswampina* (Bosc, 1802) from French Quarter Creek, Clement’s Ferry Road, Berkeley County, South Carolina at the National Museum of Natural History, Smithsonian Institution. The pigmentation has faded, but both specimens had small transverse bands (primarily in the medial region), six eye spots, and a united gonopore. In light of the findings in this study, *G.swampina* needs to be reassessed with molecular data. We conclude that *Hirudoswampina*, as described and illustrated by Bosc (1802) is *incertae sedis*.

The following redescription of the new combination *Alboglossiphoniapallida* (Verrill, 1872) is based upon the holotype of *Clepsinepallida* (YPM IZ 000253) and newly collected specimens (YPM IZ 043467–043468, YPM IZ 058354, YPM IZ 062698, YPM IZ 109351–109353, YPM IZ 106029–106030, and USNM 1662161 from New Haven County, Connecticut, USA and YPM IZ 107064 from Fairfield County, Connecticut, USA.

### ﻿Family Glossiphoniidae

#### ﻿ Alboglossiphonia pallida

Taxon classificationAnimaliaRhynchobdellidaGlossiphoniidae

(Verrill, 1872) comb. nov.

036757E8-547B-5FA9-A587-B054D7C719CD

Figs 2 , 3 , 4 , 5

##### Diagnosis.

Dark chromatophores on the dorsal surface arranged lateral to patrilaterally and medially as a thin line or interrupted thin line along with three pair of eye spots (where the first pair are closest together), six pair of crop ceca, and a united gonopore.

##### External morphology.

**Figure 3.**
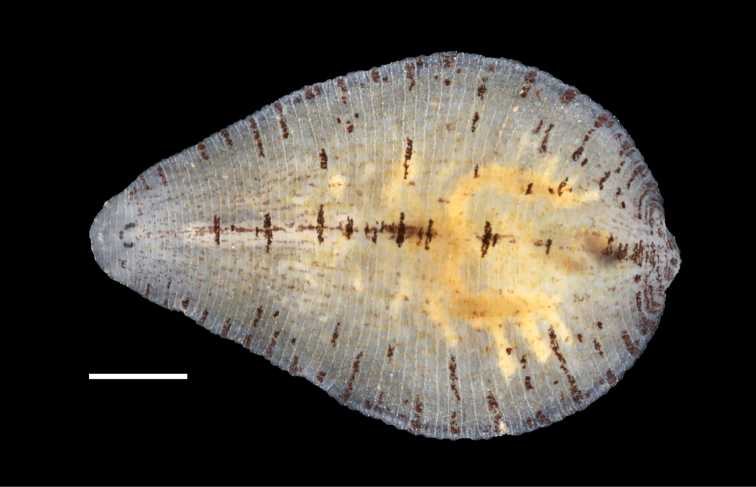
Living specimen of *Alboglossiphoniapallida* (Verrill, 1872) from the type locality of New Haven County, Connecticut, USA. YPM IZ 106029, dorsal surface Scale bar: 1 mm.

**Figure 4.**
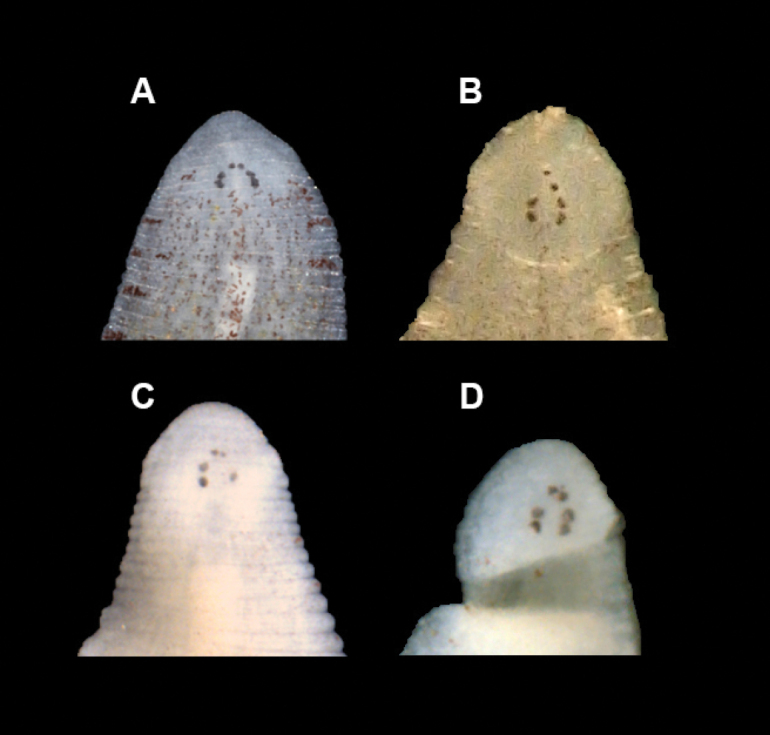
Images of the arrangement of eyespots of *Alboglossiphoniapallida* (Verrill, 1872) **A**YPM IZ 106029 **B**USNM 1662161 **C**YPM IZ 062698 **D**YPM IZ 107064.

Body narrowly ovoid to narrowly pyriform. Rounded anterior region. Dorsum buff to translucent, smooth (without papillae), and with small, black chromatophores that form thin lines with scattered areas; thin, interrupted mid-dorsal line with larger chromatophore patches (typically on sensory annuli); black chromatophores in a lateral pattern on the sensory annulus of the lateral to paralateral region (Figs 2, 3). Three pair of eye spots which are typically separate and arranged linearly or with groupings of two and four eye spots in unpigmented cephalic area with the first pair of eye spots closest together (Figs 3, 4). Some individuals have five eye spots where the first pair is present and there are only three eye spots in the second and third pair. Caudal sucker of moderate size (half diameter of mid-body) without pigment or papillae. Ventrum without pigment or papillae and with united male and female gonopores (single opening) (Fig. 2).

##### Alimentary tract.

**Figure 5.**
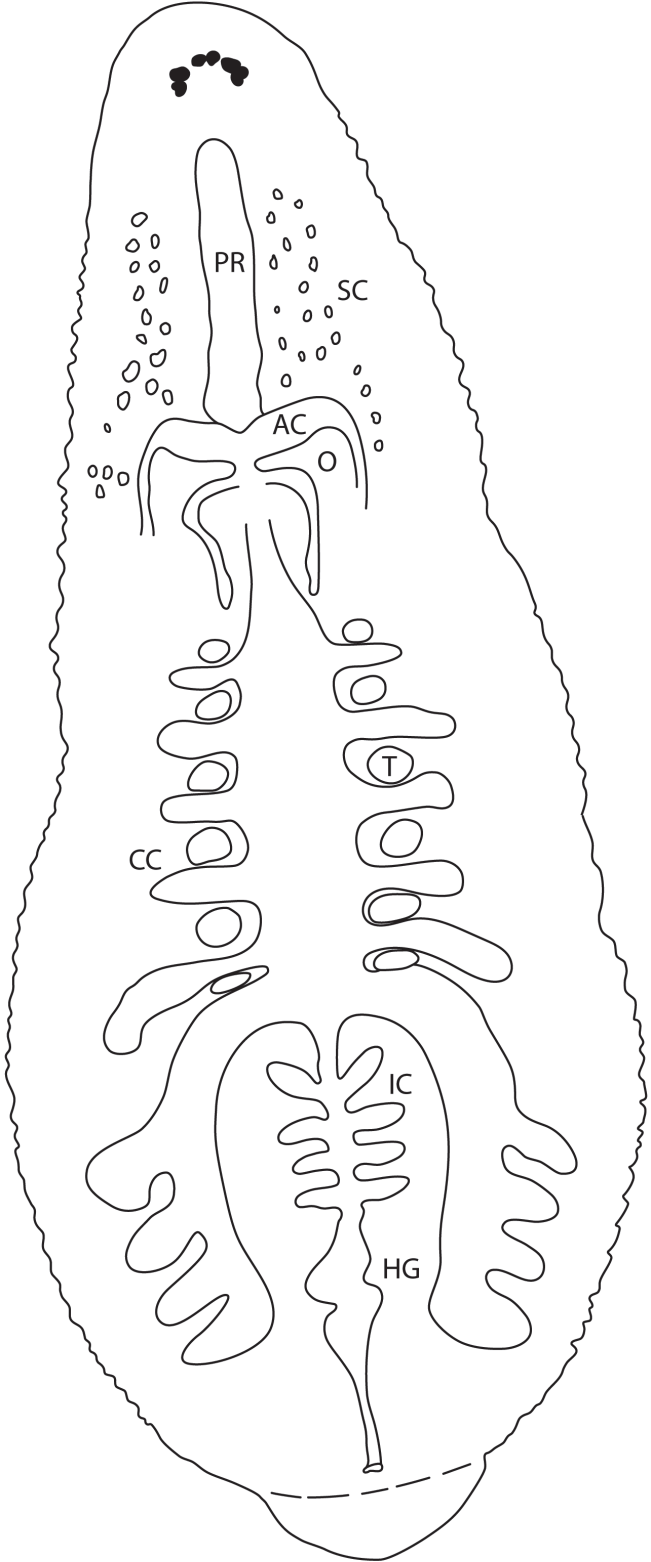
Schematic drawing of the internal morphology of *Alboglossiphoniapallida* (Verrill, 1872). Abbreviations: AC, atrial cornuae; CC, crop ceca; HG, hind gut; IC, intestinal ceca; O, ovisac; PR, proboscis; SC, salivary cells; T, testisac.

Cylindrical, blunt-tipped protrusible proboscis (approximate length of 14 annuli), opening at the center of the oral sucker. Short esophagus and diffuse salivary glands that are distributed in the anterior third of the body (Fig. 5). Crop with six pair of ceca and last pair extend posteriad and diverticulated with four sections; four pair of simple, saccular intestinal ceca with hind gut saccate and rectum opening into anus, located one annulus anteriad of the caudal sucker (Fig. 5).

##### Reproductive anatomy.

Male atrium opening into paired narrowly ovoid atrial cornua that extends laterally and narrows abruptly at junction with ejaculatory ducts and extends posteriad (Fig. 5). Six pair of testisacs between crop ceca. Pair of tubular ovisacs; length of ovisacs dependent on the reproductive state of the leech (Fig. 5). Male and female gonopores united.

### ﻿Molecular analysis

Uncorrected *p*-distances between COI sequences of each species are given in Table 2. Pairwise distances of COI sequences among *A.pallida* specimens (*n* = 5) ranged 0.24–1.05%. Among *Alboglossiphonia* species, pairwise distances of COI between *A.pallida* and specimens of *A.heteroclita* from Michigan and Wisconsin ranged 5.78–8.35%, between *A.pallida* and *A.heteroclita* (9195) from Germany ranged 12.72–12.94%, between *A.pallida* and *A.papillosa* ranged 9.07–9.7%, between *A.pallida* and *A.lata+A.weberi* ranged 10.86–13.29%, between *A.pallida* and *Alboglossiphonia* sp. 2 (MN295404) from Myanmar ranged 11.17–11.55%, between *A.pallida* and *Alboglossiphonia* sp. (MG976199) from Australia ranged 12.72–13.14%, between *A.pallida* and *A.quadrata* (AY962455) from Namibia ranged 16.84–17.1%, and between *A.pallida* and *A.iberica* (8739) from Spain ranged 17.36–17.58%.

**Table 2.** COI uncorrected pairwise sequence differences among specimens of *Alboglossiphonia* included in this study. Values presented are range followed by average in parentheses ().

	1	2	3	4	5	6	7	8	9	10
*A.iberica* (1)	—	—	—	—	—	—	—	—	—	—
*A.quadrata* (2)	17.05	—	—	—	—	—	—	—	—	—
*A.heteroclita* Germany (3)	16.47	16.13	—	—	—	—	—	—	—	—
*Alboglossiphonia* sp. S. Korea HJK (4)	15.28	14.92	11.71	—	—	—	—	—	—	—
*A.lata*/*A.weberi* (5)	15.28–17.05 (16.26)	15.21–16.96 (16.03)	13.27–14.38 (13.68)	13.18–14.44 (13.43)	0–3.96 (2.23)	—	—	—	—	—
*Alboglossiphonia* sp. 2 Myanmar (6)	14.74	16.48	14.22	13.65	6.18–7.45 (6.84)	—	—	—	—	—
*Alboglossiphonia* sp. Australia (7)	16.16	14.9	14.54	13.65	7.45–9.19 (8.19)	7.92	—	—	—	—
*A.papillosa* (8)	15.10–15.95 (15.22)	16.64–17.56 (16.77)	12.16–12.87 (12.26)	13.97–14.83 (13.97)	9.83–12.05 (10.81)	10.3–11.12 (10.3)	11.89–12.40 (11.89)	0–0.86 (0.25)	—	—
*A.heteroclita* USA (9)	16.70–17.70 (17.08)	16.32–17.71 (16.92)	11.69–12.91 (12.16)	13.38–14.75 (13.40)	10.14–13.94 (11.61)	10.94–13.11 (11.81)	12.38–14.14 (13.18)	8.40–9.77 (9.04)	0.33–3.07 (1.89)	—
*A.pallida* (10)	17.36–17.58 (17.43)	16.84–17.10 (16.95)	12.72–12.94 (12.80)	15.16–15.54 (15.31)	10.86–13.29 (11.80)	11.17–11.55 (11.35)	12.72–13.14 (12.87)	9.07–9.70 (9.24)	5.78–8.35 (6.74)	0.24–1.05 (0.73)
	1	2	3	4	5	6	7	8	9	

**Figure 6.**
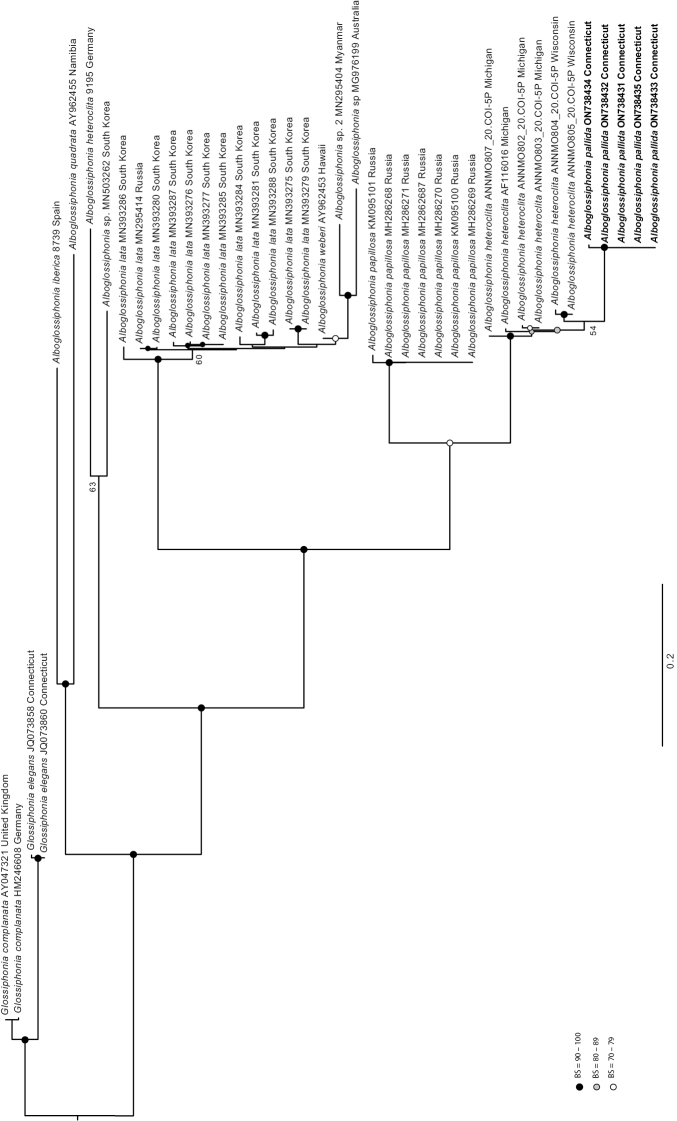
Maximum likelihood phylogeny (ln*L* = −2868.139) of *Alboglossiphoniapallida* and congeners based on mitochondrial COI sequence data partitioned by codon. Maximum likelihood bootstrap values above 70 are shown at the internodes. GenBank accession number and locality following each species name is provided for each terminal. Branches are drawn proportional to the amount of change.

The molecular dataset included COI sequences of 41 specimens (37 members of *Alboglossiphonia* and two specimens each of *Glossiphoniacomplanata* and *Glossiphoniaelegans* that served as outgroups; Table 1) and a total of 631 aligned characters. The log-likelihood of the topology was −3174.987 and the topology is shown in Fig. 6.

The genus *Alboglossiphonia* is well supported as monophyletic (bs = 100). *Alboglossiphoniapallida* and *A.papillosa* were represented by more than one individual in our analysis and each of these species was monophyletic with strong support (*A.pallida* bs = 100, *A.papillosa* bs = 98). The clade of *A.pallida* specimens (bs = 100) was adjacent to two sequences of *Alboglossiphonia* sp. from Wisconsin (bs = 54). Individuals of *A.heteroclita* were not each other’s closest relatives. *Alboglossiphoniaheteroclita* (GenBank: AF116016) from Michigan placed with three sequences of *A.heteroclita* from Michigan (BOLD:ANNMO802, BOLD:ANNMO803) and two sequences of *A.heteroclita* from Wisconsin (BOLD:ANNMO804, BOLD:ANNMO805) in a series of branches with short internodes and moderate support values (bs = 74–81). *Alboglossiphoniaheteroclita* (9195) from Germany placed sister to a sequence of *Alboglossiphonia* from South Korea (GenBank: MN503262), albeit with low support (bs = 63), suggesting these are separate species, the latter not otherwise represented in GenBank. The clade of *A.heteroclita* from Germany + *Alboglossiphonia* sp. from South Korea (MN503262) placed adjacent to clades including *A.heteroclita* from Michigan and Wisconsin, *A.pallida*, *A.papillosa*, *A.lata*, *A.weberi*, *Alboglossophonia* sp. from Myanmar, and *Alboglossiphonia* sp. from Australia (bs = 98). *Alboglossiphoniapallida* + *A.heteroclita* from Michigan and Wisconsin was sister to *A.papillosa* (bs = 75). *Alboglossiphonialata* specimens from South Korea and the specimen from Russia (MN295414) placed within a strongly supported clade (bs = 100) that included the specimen of *A.weberi* (GenBank: AY962453) from Hawaii, USA. Sequences of two unidentified specimens of *Alboglossiphonia* (MN295404 from Myanmar and MG976199 from Australia) placed sister to one another with strong support (bs = 100), within the *A.lata*/*weberi* clade, and sister to the *A.weberi* specimen from Hawaii (bs = 75). The sequences of *A.iberica* (8739) from Spain and *A.quadrata* (GenBank: AY962455) from Nambia were sister to one another (bs = 100), and that clade was well supported as sister to all other specimens of *Alboglossiphonia* in the tree (bs = 100).

Sawyer (1986) listed 14 species of the genus *Alboglossiphonia*: *A.heteroclita* (Linnaeus, 1761), *A.annandalei* Oka, 1922; *A.australiensis* (Goddard, 1908), *A.cheili* (Oosthuizen, 1978); *A.conjugata* (Oosthuizen, 1978); *A.disjuncta* (Moore, 1939); *A.intermedia* (Goddard, 1909), *A.lata* (Oka, 1910); *A.macrorhyncha* (Oosthuizen, 1978); *A.masoni* (Mason, 1974); *A.mesembrina* (Ringuelet, 1949); *A.multistriata* (Mason, 1974), ?*A.quadrata* (Moore, 1939); *A.tasmaniensis* (Ingram, 1957); and *A.weberi* (Blanchard, 1897). Subsequently, Oosthuizen (1987) redescribed *A.quadrata* (Moore, 1939) and transferred the species to the genus *Hemiclepsis*. Additionally, six more species of the genus *Alboglossiphonia* have been described, *A.polypompholyx* Oosthuizen, Hussein, and El-Shimy, 1988; *A.disuqi* El-Shimy, 1990; *A.pahariensis* Nesemann & Sharma, 2007; *A.kashiensis* Nesemann, 2007; *A.iberica* Jueg, 2008; *A.levis* Gouda, 2010 and five additional species have been elevated or resurrected, *A.hyalina* (O.F. Müller, 1774); *A.inflexa* (Goddard, 1908); *A.papillosa* (Braun, 1805); *A.novaecaledoniae* (Johansson, 1918); *A.striata* (Apáthy, 1888). The species *Clepsinepallida* is herein resurrected in the new combination *Alboglossiphoniapallida* (Verrill, 1874), thus, making 25 recognized species of the genus *Alboglossiphonia*.

*Alboglossiphoniapallida* was strongly supported by morphological and molecular evidence as a species within the genus *Alboglossiphonia* and distinct from North American *A.heteroclita*, European *A.heteroclita*, *A.lata*, *A.weberi*, *A.iberica*, and *A.papillosa*. *Alboglossiphoniapallida* is characterized by having dark chromatophores on the dorsal surface arranged lateral to patrilaterally and medially as a thin line or interrupted thin line along with three pair of eye spots (where the first pair are closest together, the defining characteristic of the genus *Alboglossiphonia*), six pair of crop ceca, and a united gonopore. The non-monophyly of *A.heteroclita* continues to pose a challenge. The *A.heteroclita* specimens from North America were 11.69–12.91% different from *A.heteroclita* from Europe, indicating that the North American specimens are not *A.heteroclita* and most likely represent an undescribed species. The *A.heteroclita* specimens from North America were 0.33–3.07% different from one another. These specimens form a strongly supported clade with *A.pallida* (bs = 100), although the North American *A.heteroclita* specimens as a group were 5.78–8.35% different from the *A.pallida* specimens. North American specimens assigned to *A.heteroclita* are typically characterized by a lack of pigmentation on translucent bodies (Sawyer 1972, 1973; Klemm 1982; Moser et al. 2016). In North America, A.cf.heteroclita has been reported in the Great Lakes region and as far west as Nebraska in the USA and as far west as British Columbia in Canada (Klemm 1982; Moser 1991). Further collection is needed to elucidate the taxonomy and geographic distribution of the North American *Alboglossiphonia* specimens that were identified as *A.heteroclita* and its relationship with *A.pallida*.

In Europe, *A.heteroclita* had been a heterogenous concept and known more by the infraspecific varieties. These synonymies have since been elevated to the species rank with *A.hyalina* (O.F. Müller, 1774) having yellow chromatophores and no dark chromatophores and *A.striata* (Apáthy, 1888) with dark, transverse pigmentation (Lukin 1976; Nesemann and Neubert 1999; Jueg and Grosser per. comm.). As described and figured by Braun (1805), *A.papillosa* (Braun, 1805) has dark medial spots and some scattered dark chromatophores. This description is consistent with the description of *A.heteroclita* (Linnaeus, 1761) (Nesemann and Neubert 1999; Jueg and Grosser per. comm.). *Alboglossiphoniaheteroclita* from Germany was 12.16–12.87% different than *A.papillosa* collected from Russia. As photographed by Klass et al. (2018), the specimen of *A.papillosa* has dark dorsal lines and is potentially a previously undescribed species.

Sequences of specimens from Asia, Australia, and Hawaii form a strongly supported clade, except for a single sequence from South Korea (MN503262). Sequences of *A.lata* form a clade with short internodes that were poorly supported for the most part. The clade predominantly consisted of sequences from South Korea, yet also included a single sequence from Primorsky Krai, Russia (MN295414) and the sequence of *A.weberi* from Hawaii, USA (AY962453). *Alboglossiphonialata* is a widely distributed species that is considered invasive and spread via the aquatic plant trade. In particular, the specimen of *A.weberi* from Hawaii should be reexamined to determine if this might be an occurrence record of the invasive *A.lata*, which would be concerning for the Hawaiian island ecosystem. The sequences of *Alboglossiphonia* from Myanmar and Australia are supported as members of the genus and likely represent species distinct from one another and not otherwise represented in this analysis or publicly available databases (e.g., GenBank, BOLD), yet the specimens need to be examined to determine the species identification as there have been seven described species from Australasia and Oceania.

The sequence of *Alboglossiphoniaquadrata* (AY962455) from Namibia has likely been assigned the incorrect name. Oosthuizen (1987) transferred the species name *quadrata* to the genus *Hemiclepsis*. This sequence is highly supported as a lineage within *Alboglossiphonia* and the specimen needs to be reexamined to determine if it belongs to one of the seven species of *Alboglossiphonia* described from Africa (Gouda 2010).

In this study, COI was largely successful at distinguishing congeners of *Alboglossiphonia*, but it had limited utility in resolving the relationships between species. Combining COI data with other loci, especially nuclear loci, is needed to determine relationships between glossiphonid species with confidence. The addition of sequences of more *Alboglossiphonia* species will improve our understanding of relationships within the genus. This study included all publicly available *Alboglossiphonia* sequences, although this represents only about one-third of the diversity of the genus.

## ﻿Conclusion

*Alboglossiphoniapallida* (Verrill, 1872) is resurrected and redescribed based on morphological and molecular data that demonstrate it is distinct from the specimen assigned to *A.heteroclita* from Michigan and Wisconsin and *A.heteroclita* from Europe, as well as other species of *Alboglossiphonia*. Additional sampling of *Alboglossiphonia* is needed to understand its phylogeny especially as many species have not been collected since their original description.

## Supplementary Material

XML Treatment for Alboglossiphonia pallida
